# A Novel Insight into the Identification of Potential SNP Markers for the Genomic Characterization of Buffalo Breeds in Pakistan

**DOI:** 10.3390/ani13152543

**Published:** 2023-08-07

**Authors:** Muhammad Anas, Muhammad Farooq, Muhammad Asif, Waqas Rafique Ali, Shahid Mansoor

**Affiliations:** 1 National Institute for Biotechnology and Genetic Engineering (NIBGE), Faisalabad 38000, Punjab, Pakistan; 2 Department of Animal Sciences and Center for Nutrition and Pregnancy, North Dakota State University, Fargo, ND 58105, USA

**Keywords:** breed-specific markers, *Bubalus bubalis*, buffalo genomics, buffaloes, Nili, selection signatures, SNPs

## Abstract

**Simple Summary:**

Buffaloes, as a form of livestock, have a huge economic impact, especially in the Asiatic market. In Pakistan, the production of buffaloes for either their milk or meat has increased beyond the overall level of cattle production. The domestication of Nili–Ravi buffaloes from the Nili and Ravi breeds and their selection for their milk production also indicate the economic potential of buffalo breeds in Pakistan. However, very limited genetic information is available for their characterization. Therefore, to identify the selection markers relevant to Pakistani buffalo breeds, available genome sequences of Pakistani buffalo breeds and buffalo breeds from 13 other countries were retrieved from our previously published study. These data were then analyzed through our developed pipeline for potential breed-specific markers. The novel aspect of this pipeline is that it compares single-nucleotide polymorphisms (SNPs) in the same position for possible allele differences among breeds. This approach is different from other currently available tools, which select SNPs based on whether their position is present or absent in one breed compared with others. This study also highlights how the Nili breed, which was previously thought to be lost due to Nili–Ravi domestication, has maintained its separate breed status and requires a breed conservation program.

**Abstract:**

Domestic buffaloes (*Bubalus bubalis*), known as water buffaloes, play a key role as versatile multipurpose agricultural animals in the Asiatic region. Pakistan, with the second-largest buffalo population in the world, holds a rich domestication history of buffaloes. The overall trends in buffalo production demand the genomic characterization of Pakistani buffalo breeds. To this end, the resequencing data of Pakistani breeds, along with buffalo breeds from 13 other countries, were retrieved from our previous study. This dataset, which contained 34,671,886 single-nucleotide polymorphisms (SNPs), was analyzed through a pipeline that was developed to compare possible allele differences among breeds at each SNP position. In contrast, other available tools only check for positional SNP differences for breed-specific markers. In total, 1918, 1549, 404, and 341 breed-specific markers were identified to characterize the Nili, Nili–Ravi, Azakheli, and Kundi breeds of Pakistani buffalo, respectively. Sufficient evidence in the form of phenotypic data, principal component analysis, admixture analysis, and linkage analysis showed that the Nili breed has maintained its distinct breed status despite sharing a close evolutionary relationship with the Nili–Ravi breed of buffalo. In this era of genome science, the conservation of these breeds and the further validation of the given selection markers in larger populations is a pressing need.

## 1. Introduction

Agriculture is a major contributor to livelihood in both developed and lesser-developed countries. The livestock sector is considered the backbone of agricultural countries such as Pakistan [1]. Its contribution to human health is indispensable as it is a source of economical high-quality proteins either in the form of milk or meat. Among livestock species, buffaloes are the foremost source of milk, contributing to about 60% of the total milk produced in Pakistan [2] and 55% of the total milk produced in India [3]. However, there is still a need for genomic selection to close the gap between the production of and demand for milk. According to the US dietary guidelines, an adult should consume 732 mL (3 cups) of milk per day [4], but in Pakistan, the per-day availability of milk is about 608 mL [5]. These facts and figures are estimated considering individual daily consumption, and the statistics can be astonishing if amplified and analyzed annually considering the overall population of Pakistan, which is about 220 million.

The improvement of livestock through genomic selection is thus required to cope with this demand [6,7]. Moreover, in this era of buffalo genome science, the characterization and selection of buffalo breeds is a pressing need [8], especially in underdeveloped countries. The buffaloes of the Asiatic region can be classified as river- or swamp-type buffalo, depending upon their history of domestication [9]. Swamp-type buffaloes are specialized for draught purposes, while river-type buffaloes are specialized for milk production [10]. All five breeds of Pakistan, the Nili, Ravi, Nili–Ravi, Kundi, and Azakheli breeds, are river-type buffaloes and are known globally for their milk production [11]. The Nili, Nili–Ravi, and Ravi breeds are native to the Punjab Province of Pakistan and are typically found in regions near the Sutlej River, the districts of central Punjab, and the Ravi River, respectively [11,12]. The Kundi breed is most specifically found in Sindh Province on the banks of the Indus River, while the Azakheli breed can be located in the Swat district of Pakistan’s KPK Province [12].

Genetic breed characterization involving breed composition, pedigree, and parentage analyses was traditionally carried out through microsatellite-based markers [13,14], but currently, single-nucleotide polymorphism (SNP) markers have replaced microsatellites by proving their potential [15,16,17,18]. Several studies to date have shown the implications of SNPs in the breed-specific identification of individuals, specifically in cattle populations [19,20]. However, researchers are still comparing different SNP-based approaches with microsatellite-based methods for better breed traceability and panel development in cattle [15,21] and, per a recent report, in pigs [22]. Given these scenarios in the cattle and pig industry, we developed an alternate approach to identify breed-specific markers in buffalo, which were then tested against the sequencing data available for Pakistani breeds.

The pipeline we developed works by comparing possible allele differences among breeds at SNP positions, while other available tools only check for positional SNP differences or clustering based on SNP positions for breed-specific markers. We hypothesized that even though the same SNP positions can be present in different closely related breeds, their allelic differences can nevertheless separate them into different breeds. Based on our hypothesis, a few animals of the Nili breed, considered to be lost during the process of domesticating the Nili–Ravi breed [23], were identified. We then verified these findings through the phenotypic characterization of buffalo from remote areas of Pakistan, such as Okara, Sahiwal, and Pakpattan. It was found that some well-established farmer families in these areas were not only conserving the Nili animals but were also using conventional breeding techniques to enhance their overall production. 

**Figure 1:**
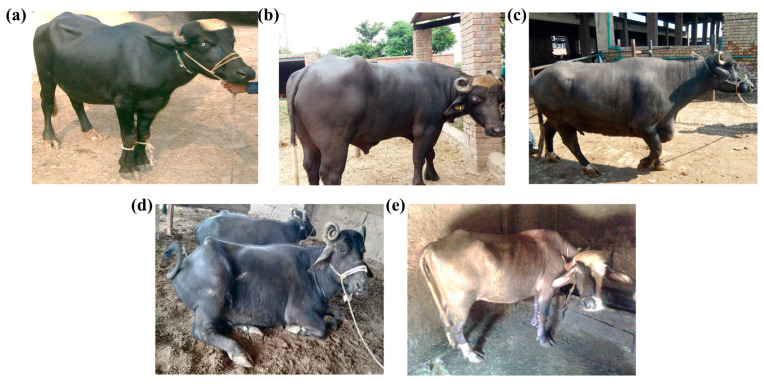
Buffalo breeds of Pakistan showing typical phenotypic markings of (**a**) the Nili breed (**b**) the Nili–Ravi breed (**c**) the Ravi breed (**d**) the Kundi breed, and (**e**) the Azakheli breed.

With the reappearance of the Nili breed, we found that the use of phenotypic characterization alone was insufficient to differentiate the Nili–Ravi breed from the Nili breed. The Nili–Ravi breed is typically recognized based on its five typical markings, known as punj kalyan [3]. However, these markings can also be seen in the Nili breed (see Figure 1). Therefore, in such scenarios, our developed approach based on SNPs can serve as a turning point for the characterization of buffalo breeds from Pakistan. Moreover, this can provide an efficient selection tool, compared with the microsatellite-based approach previously tested [24].

## 2. Materials and Methods

### 2.1. Phenotypic Data Collection

The phenotypic data of Nili and Nili–Ravi were collected from five different areas of Pakistan, i.e., Faisalabad, Chichawatni, Pakpattan, Lahore, and Qadirabad. Different phenotypic parameters were considered for the analysis of these dairy animals. No buffalo-specific standard guidelines are available for phenotypic data markings. Consequently, in this study, the conformational guidelines of the International Committee for Animal Recording (ICAR) for dairy cattle [25] were used to collect phenotypic records for Nili and Nili–Ravi breeds. Nine parameters were taken into consideration for males, and eleven parameters were noted for females for phenotypic characterization. Live body weight was calculated based on Schaeffer’s formula [26] as follows:

$$W=\frac{l\times g^{2}}{300}$$

where ‘W’ is live body weight in pounds (lbs), ‘l’ represents the animal length (inches) from the pin bone to the shoulder bone, and ‘g’ is the heart girth (inches) of the animal. Other phenotypic parameters of male buffalo collected using ICAR guidelines were stature, chest width, body depth, length, girth, rump width, face length, and scrotal circumference. In the case of females, the measures were stature, chest width, body depth, length, girth, rump width, fore teat length, rear teat length, rear udder width, and teat thickness. 

### 2.2. Data Retrieval and Preprocessing

The genotypic data of Pakistani breeds, along with breeds from 13 other countries, were retrieved from the results of a collaborative study of our lab [10] and then grouped based on river- and swamp-type breeds. The SNP data were procured for all 228 individuals (river type = 97, swamp type = 131; defined below) each containing 34,671,886 variants from given countries.

- River type = Pakistan (*n* = 18), India (*n* = 3), China (*n* = 23), Nepal (*n* = 5), Italy (*n* = 13), Iran (*n* = 20), Iraq (*n* = 9), and Bengal (*n* = 6);
- Swamp type = China (*n* = 99), Vietnam (*n* = 5), Philippines (*n* = 5), Laos (*n* = 2), Burma (*n* = 5), Indonesia (*n* = 10), and Thailand (*n* = 5).

The variant data were organized for Nili (*n* = 4), Nili–Ravi (*n* = 4), Azakheli (*n* = 5), Kundi (*n* = 5), and breeds of other countries based on BCFtools [27]. 

### 2.3. Principal Component Analysis (PCA)

Principal components analysis was performed using PLINK [28] to identify the clustering of breeds within the river- and swamp-type breeds based on SNP variation. Furthermore, PCA was performed among river-type breeds and then specifically for Pakistani breeds to assess the extent of genetic variation among individuals. The PCA score and the graphs were plotted using R v4.1.2 [29].

### 2.4. Admixture Analysis

Admixture analysis of buffalo breeds from Pakistan was performed in comparison to other river-type breeds using ADMIXTURE [30] at different levels of *k*, assuming population/genetic groups with values ranging from 2 to 11 using the default quasi-Newton convergence method. The data were then plotted against the derived *k* values using R v4.1.2 [29] to assess the extent to which these population groups can be isolated based on hierarchy. This approach previously has been adopted to characterize individuals to their population of origin based on hierarchy [31], so we tested for Pakistani breeds to validate their breed status.

### 2.5. Linkage Disequilibrium (LD) Decay Analysis

The pairwise LD decay analysis of the buffalo breeds of Pakistan was conducted using a genotypic correlation coefficient (*r*^2^) using PopLDdecay [32], with a window size of 0.1 Mb. The results were then plotted using a script provided with the PopLD decay package. LD analysis was conducted to determine the diversity among Pakistani breeds, especially between Nili and Nili–Ravi breeds, and to verify the appearance of four characterizable breeds, as indicated in PCA and admixture analyses.

### 2.6. Identification of Breed-Specific Markers (BSMs) and Annotation

**Figure 2:**
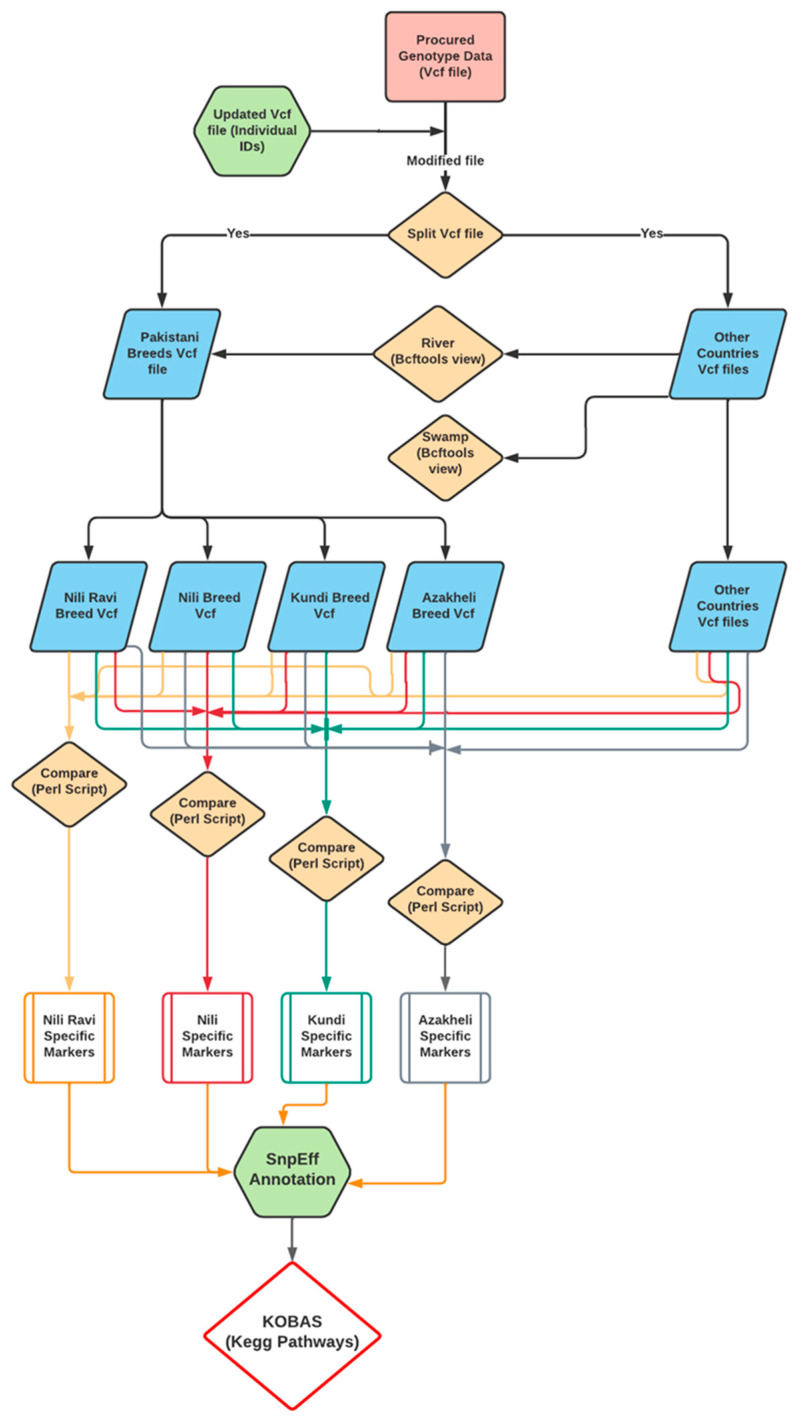
Pipeline for identification and characterization of breed-specific markers (BSMs) of the Nili–Ravi, Nili, Kundi, and Azakheli breeds using resequencing genotypic data.

Breed-specific markers were identified using a custom Perl language script, provided in Supplementary Data S6. The script identifies the SNPs specific to a breed if that unique genotype allele specifically exists in all samples of the same breed with the relaxation of one sample. The relaxation of one sample was due to the close evolutionary history of the Nili and Nili–Ravi breeds. Finally, the identification of the breed-specific variants was carried out after comparing them against the rest of the Pakistani breeds as well as against all river- and swamp-type buffaloes from the 13 different countries. The pipeline used to identify the BSMs is shown in Figure 2.

The BSMs were then annotated using SnpEff [33] against the reference river-type genome [10] to obtain the gene sequences involved in these markers. These gene sequences were further annotated and tested for over-represented KEGG pathways through KOBAS [34], and they were then compared against water buffalo reference data.

## 3. Results

### 3.1. Phenotypic Characterization

**Figure 3:**
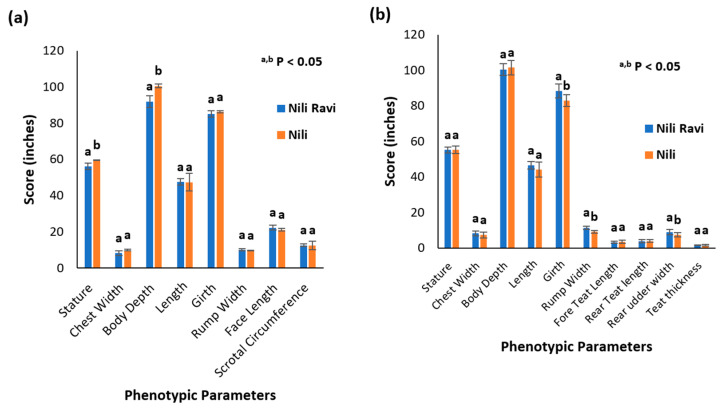
Phenotypic characterization of Nili and Nili–Ravi buffalo breeds: (**a**) comparison by means of phenotypic parameters in Nili and Nili–Ravi adult males, where ^a,b^ *p* < 0.05 represents mean ± SD, with different superscript differentiating based on *t*-test statistics; (**b**) comparison by means of phenotypic parameters in Nili and Nili–Ravi adult females, where ^a,b^ *p* < 0.05 represents mean ± SD, with different superscript differentiating based on *t*-test statistics.

Determining breed characterization based on phenotypic parameters is a conventional approach. Kundi and Azakheli breeds of Pakistan are describable based on their typical phenotypic parameters and can be differentiated to some extent based on the presence or absence of characteristic markings [12]. The appearance of the Nili breed complicates the characterization and differentiation of Pakistani buffalo breeds based on phenotypic parameters only. This is particularly true for the Nili and Nili–Ravi breeds. Therefore, to compare the Nili and Nili–Ravi breeds purely based on phenotypic parameters, different measurements were compared using a two-tail *t*-test. The records are presented in a tabulated form in Supplementary Data S1 for a better understanding of Figure 3.

In the case of Nili, adult body weight was 532 ± 62 kg and 458 ± 52 kg for males and females, respectively. In comparison, in the Nili–Ravi breed, the adult body weight calculated was 519 ± 30 kg and 551 ± 67 kg for males and females, respectively. 

### 3.2. Breed Clustering Using Genomic Markers

**Figure 4:**
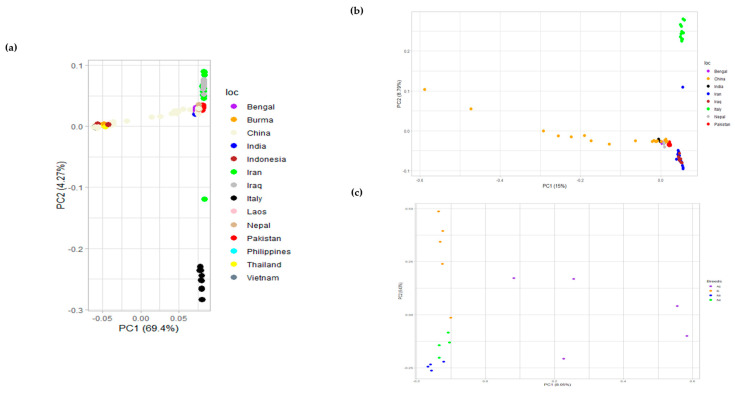
(**a**) Principal component analysis (PCA) of river- and swamp-type buffalo breeds with clustering that separates common buffalo breeds of Iran and Italy; (**b**) PCA of river-type breeds only showing clustering that separates breeds of Italy, Iran, and Iraq from the rest of the river-type breeds; (**c**) PCA of buffalo breeds of Pakistan, i.e., Azakheli (Az), Kundi (K), Nili (Ni), and Nili–Ravi (Nr).

Principal component analysis (PCA) explains the clustering of different breeds based on covariance. The first two principal components (PCs) explaining most of the variance were plotted using *R*, for swamp- and river-type breeds. Furthermore, PCA for river-type and only Pakistani breeds was plotted to observe their patterns, as shown in Figure 4. The patterns revealed a clear separation between the Nili and Nili–Ravi breeds and a close association between the two was observed. This indicated that there could potentially be some differences between these two breeds that need to be further investigated on a genomic level. 

### 3.3. Admixture and Linkage Disequilibrium (LD) Decay Analysis

**Figure 5:**
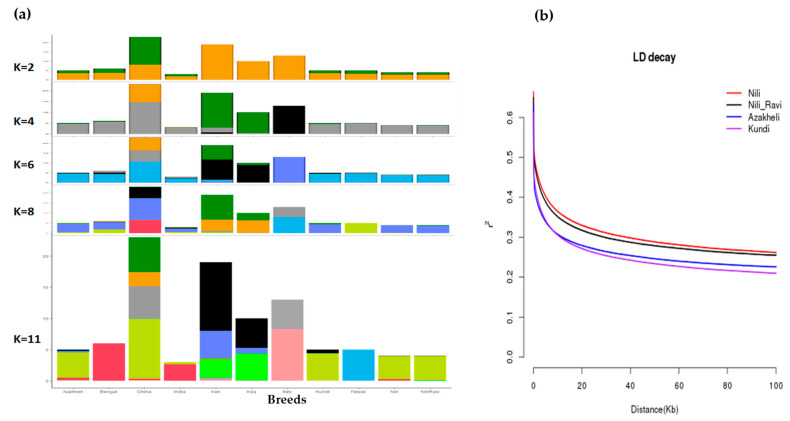
(**a**) Admixture analysis of all river-type buffalo breeds using ADMIXTURE at population levels of 4, 6, 8, and 11, where the sharing of colors corresponds with sharing of germplasm among breeds; (**b**) LD decay analysis of buffalo breeds of Pakistan (Nili, Nili–Ravi, Kundi, and Azakheli) using PopLDdecay package at a window size of 0.1 Mb.

To find the origin of Pakistani breeds, especially Nili and Nili–Ravi, compared with the rest of the river-type buffaloes, allelic admixture analysis was used. The results of the admixture analysis against different *k*-levels, assuming 2 to 11 population/genetic groups, are shown in Figure 5a. The results were further validated through linkage disequilibrium, identifying the nonrandom association of alleles at two or more loci of the population. To determine the diversity among buffalo breeds in Pakistan, LD decay analysis was conducted, and the results are shown in Figure 5b. The LD decay results with a spanning window of 0.1 Mb clearly indicate Nili as a separate and the most uniform breed among the four breeds, whereas the Kundi breed was the most diverse among the investigated breeds.

### 3.4. Identification of Breed-Specific Markers (BSMs)

The identification of breed-specific markers for breeds within a specific region is the most important step toward the characterization of buffaloes from that region. The BSMs were identified through the Perl language script, which is provided in Supplementary Data S6. This script was written to compare Pakistani breeds at the genotypic level with all the other mentioned river- and swamp-type buffaloes. The pipeline used to identify BSMs was previously shown in Figure 2. 

**Figure 6:**
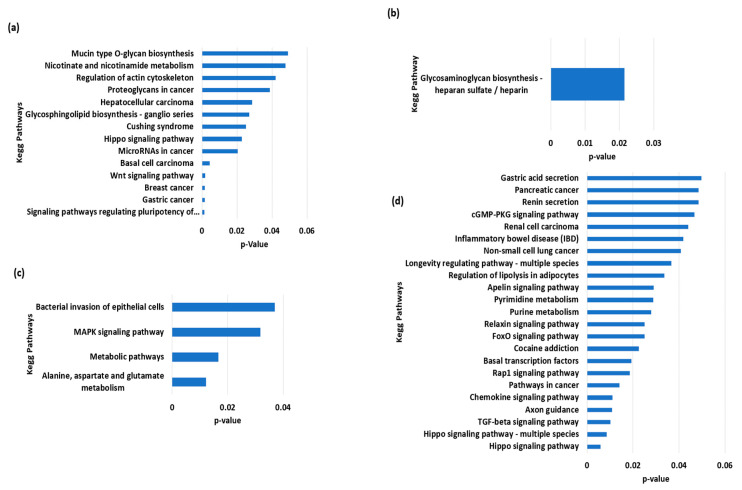
Functional over-represented KEGG pathways associated with genes including breed-specific markers of buffalo breeds of Pakistan: (**a**) Azakheli, (**b**) Kundi, (**c**) Nili, and (**d**) Nili–Ravi.

The total variants were about 34,671,886, while those that were unique to Nili, Nili-Ravi, Azakheli, and Kundi were 1918, 1549, 404, and 341, respectively. The VCF (variant call format) files of BSMs for each of the given Pakistani breeds are provided in Supplementary Materials S2–S5 along with Figures S1–S4, presenting the annotated summary of BSMs of Nili, Nili–Ravi, Azakheli, and Kundi, respectively. The identified BSMs were annotated using SnpEff, and then the genes associated with BSMs, given in annotated VCF files, were analyzed using KOBAS to determine the over-represented KEGG pathways (Figure 6). The over-represented pathways we identified exhibited selection for traits associated with energy metabolism and longevity in Nili and Nili–Ravi breeds, respectively. Moreover, the pathways associated with cancer were found in the Azakheli breed, while those associated with immunity were found in the Kundi breed.

## 4. Discussion

As an agricultural subsector, livestock plays an essential role in providing human food supplies worldwide. However, addressing malnutrition and food security are still global challenges [35]. Increasing the efficiency of animal agriculture will help improve food security. In Pakistan, a barrier to improving the efficiency of animal production and thus improving human food security is that the genetic potential of indigenous livestock breeds has not been adequately determined. Consequently, the characterization of buffalo breeds is an important step [36]. Currently, the Nili–Ravi, Kundi, and Azakheli breeds are considered to be the only buffalo breeds of Pakistan as the Nili and Ravi breeds are considered to have been lost during the process of domestication of Nili–Ravi [23]. However, in this study, a few families of farmers were identified in remote areas of Pakistan, namely Pākpattan and Okara, who were not only preserving the Nili breed, but the animals were in a continuous process of selection for production traits over time. 

Phenotypic parameters were collected according to ICAR guidelines as this is one of the conventional approaches based upon which Nili can be differentiated from the rest of the buffalo breeds, especially from Nili–Ravi. The results indicate that the Nili breed is not significantly different from Nili–Ravi in terms of phenotypic parameters. Nili was considered a short-statured breed, known for its desirable appearance traits, while phenotypic results indicate that the stature and body depth of Nili bulls are significantly greater than Nili–Ravi bulls. However, no differences were observed in female buffalos. Similarly, female phenotypic records document significant differences in girth, rump width, and rear udder width. So, based on these results, we conclude that it is difficult to differentiate these breeds in terms of phenotypic data only. Small sample sizes can also be the reason for the undifferentiable phenotypic data observed in this study.

For the genotypic characterization of breeds, PCA was conducted to assess the diversity among buffalo breeds as tested before in tracing SNPs in cattle breeds [37]. Evident clustering among river- and swamp-type breeds was observed (Figure 4a), although the diversity observed in Chinese breeds was obvious because most are crossbreeds of swamp-type breeds from China and river-type breeds from the Indo-Pak region. The PCA results of a previous study [10] also revealed a similar clear clustering among river- and swamp-type breeds but was more focused toward swamp-type breeds. To gain a better understanding of how diverse the river-type and Pakistani breeds are, the results were separately plotted (Figure 4). Although the Pakistani breeds are very similar when compared to the rest of the river-type breeds, they could be differentiated when plotted separately. Among river-type breeds, the breeds of Iran and Iraq were separately clustered, as shown in Figure 4a. Italian Mediterranean buffaloes were differentiated from both river- and swamp-type breeds, supporting the hypothesis that these buffaloes were never bred with animals of other breeds after their domestication from Egypt to Italy [38]. However, river-type breeds are considered to be originated from the Indo-Pak region and to have spread to Italy via Iran, Egypt, and Eastern Europe, while swamp types are considered to be originated from Thailand and then to have spread to the Islands of Indonesia via China and the Philippines [9]. Such domestication events were also highlighted in the PCA of Pakistani breeds, suggesting some diversity among Azakheli and Kundi animals, while Nili appeared to be the most uniform among the four breeds (Figure 4c). Similarly, samples of the Nili breed revealed a close similarity to the Nili–Ravi breed. Since the variance was not well explained in PCA results for river-type breeds, the multidimensional scaling (MDS) analysis was performed for validation to interpret pair-wise distances and clustering based on the identity-by-state (IBS) method in cartesian space. The results of MDS analysis further supported PCA results, as shown in Figure S1 of Supplementary Materials.

PCA clustering results and the identification of Nili as a separate breed were further confirmed using admixture and linkage disequilibrium analyses, respectively. Admixture ancestry analysis, suggested by Libiger and Schork [39], was also carried out at different *k*-levels to find the extent to which germplasm was shared among river-type breeds and to study the origin of Pakistani breeds, especially the Nili–Ravi breed, with respect to the Nili breed. At *k* = 2, the clustering pattern revealed differentiation among the breeds originating from Iran, Iraq, and Italy, while the rest were clustered. At *k* = 6–8, the PCA pattern of river-type breeds revealed that breeds from Iran and Iraq shared germplasm, while Italian breeds were separated. At *k* = 11, Pakistani breeds were separated and exhibited shared germplasm, whereas breeds from Nepal and Italy revealed distinct patterns. Since Pakistani breeds appeared to share germplasm, the extent of linkage disequilibrium (LD) was then measured for Pakistani breeds to determine how distinct these breeds are from each other [40]. The extent of linkage disequilibrium (*r*^2^) decay of Nili compared with Nili–Ravi, Azakheli, and Kundi can be observed in Figure 5b. The most extensive LD was observed in the Nili breed, while the least was observed in the Kundi breed, at a window distance of 0.1 Mb. The rapid decay of LD in Kundi indicates that it is the most diverse among the four breeds, followed by Azakheli, Nili–Ravi, and Nili, respectively. 

After the validation of the breed status of Pakistani buffaloes, breed-specific markers were identified using Perl language script (Supplementary Data S6), as no other tool was available to compare a specific breed to the others at the allelic level. Furthermore, as the samples were compared based on allelic differences, the positions that carried similar alleles to all samples of the same breed were selected, but based on the PCA and MDS analyses results presented here, the relaxation of one sample was provided. One sample of the Nili and Kundi breeds demonstrated clustering with Nili–Ravi, so this sample was considered in the script, but we also provide a script in Supplementary Data S7 without exception to any such conditions for future use. These BSMs were mostly intergenic (Figures S2–S5), which indicates that there is a certain number of genes based upon which these breeds can be characterized. 

The genes involving BSMs were subjected to KEGG pathway enrichment to determine how these breeds were selected during domestication. The results revealed that almost all Pakistani breeds are adaptive to the country’s harsh climatic conditions, which is why most of the enriched pathways are involved in immunity, cell death, and neural and cancerous diseases. The pathways associated with the Nili–Ravi and Nili breeds indicate their selection for traits associated with growth (MAPK signaling and TGF-beta signaling) and longevity, as well as nucleotide and amino acid metabolism. The Kundi breed exhibited enrichment for glycosaminoglycan biosynthesis via the heparin sulfate/heparin pathway, which is known to be involved in immunity [41]. In comparison, the Azakheli breed revealed significant enrichment for pathways associated with cancerous diseases, primarily due to its coat color and because it is kept in the dark to avoid these diseases [42], as shown in Figure 1e. However, further genetic selection for production traits is needed in these breeds to enhance their productivity and further explore their genetic potential.

Genotypic and phenotypic data indicate that the animals of Nili origin still maintain their distinct breed status. Buffalo Breeders Association has also kept records of pure-bred animals for further characterization and validation of these BSMs at the mass level, either by designing Kompetitive allele-specific PCR (KASP) markers or through the low-depth sequencing of these genes [43]. The implementation of characterization, selection, and conservation programs by the government can assist farmers not only through the maintenance of pure germplasm but also by improving livestock production.

## 5. Conclusions

This study provides the basis for the re-emergence of the Nili breed along with the potential markers of genotypic characterization for major buffalo breeds of Pakistan. This is the first study of its nature where genetic signatures were identified using whole-genome resequencing data by studying the SNP-based allelic differences among buffalo breeds. It was difficult to distinguish the Nili–Ravi breed from the Nili breed based on phenotypic parameters only, as both breeds have similar markings. Therefore, this genotype-based approach will provide more accurate information to the Buffalo Breeders Association for validating its records further. Genotypic and phenotypic data provide evidence of a history of shared domestication between the Nili and Nili–Ravi breeds, which is further supported by the results of admixture, LD decay, and BSM analyses presented here. Admixture analysis revealed that although all Pakistani breeds share germplasm due to their shared domestication, they still hold a distinct breed status. The results of LD decay analysis then confirmed this finding by clearly providing evidence of four separate breeds based on the given samples. All these analyses designate the Nili breed as a more homogenized and uniform breed than the Nili–Ravi and Azakheli breeds. However, Kundi appeared to be the most diverse breed. Although the selection signatures for the characterization of these breeds were identified, there is a need for further validation at a mass level using strategies like KASP and whole-genome resequencing with relatively low coverage depth (5–10X). The pathway enrichment analysis for KEGG pathways revealed that Nili and Nili–Ravi have been previously selected for production traits during domestication, but for enhanced productivity, there is a need for aggressive selection and preservation programs for all these breeds. The approach developed here should be tested in the genotypic characterization of all species, specifically those related to the livestock sector, for validation and better selection based on SNP markers.

## Data Availability

The data presented in this study are available in Supplementary Materials.

## Supplementary Materials

The following supporting information can be downloaded at: https://www.mdpi.com/article/10.3390/ani13152543/s1, Figure S1: (a) Multidimensional scaling (MDS) analysis of river- and swamp-type buffalo breeds showing IBS clustering for river- and swamp-type breeds from Iran and Italy; (b) MDS analysis of river-type breeds only showing IBS clustering for Italy, Iran, Iraq and rest of the river-type breeds; (c) MDS analysis of buffalo breeds of Pakistan; Figure S2: Annotated summary of SNPs involving the Nili breed’s specific markers through SnpEff using river-type water buffalo genome; Figure S3: Annotated summary of SNPs involving the Nili–Ravi breed’s specific markers through SnpEff using river-type water buffalo genome; Figure S4: Annotated summary of SNPs involving the Azakheli breed’s specific markers through SnpEff using river-type water buffalo genome; Figure S5: Annotated summary of SNPs involving the Kundi breed’s specific markers through SnpEff using river-type water buffalo genome; Data S1: Phenotypic parameters of Nili and Nili–Ravi breeds recorded separately for male and female animals to differentiate them phenotypically; Data S2–S5: Breed-specific marker VCF files for Nili, Nili–Ravi, Azakheli, and Kundi breeds of Pakistan; Data S6–S7: Perl scripts for identification of breed-specific markers modified as per our sample criteria and without modification.
