# Supplementary material for: A Novel Insight into the Identification of Potential SNP Markers for the Genomic Characterization of Buffalo Breeds in Pakistan
Source: Animals (Basel). 2023 Aug 7;13(15):2543. doi: 10.3390/ani13152543 (PMC10416883; doi:10.3390/ani13152543)
Supplement: Supplementary file 1 [file animals-13-02543-s001.zip › Supplementary Figures.docx]

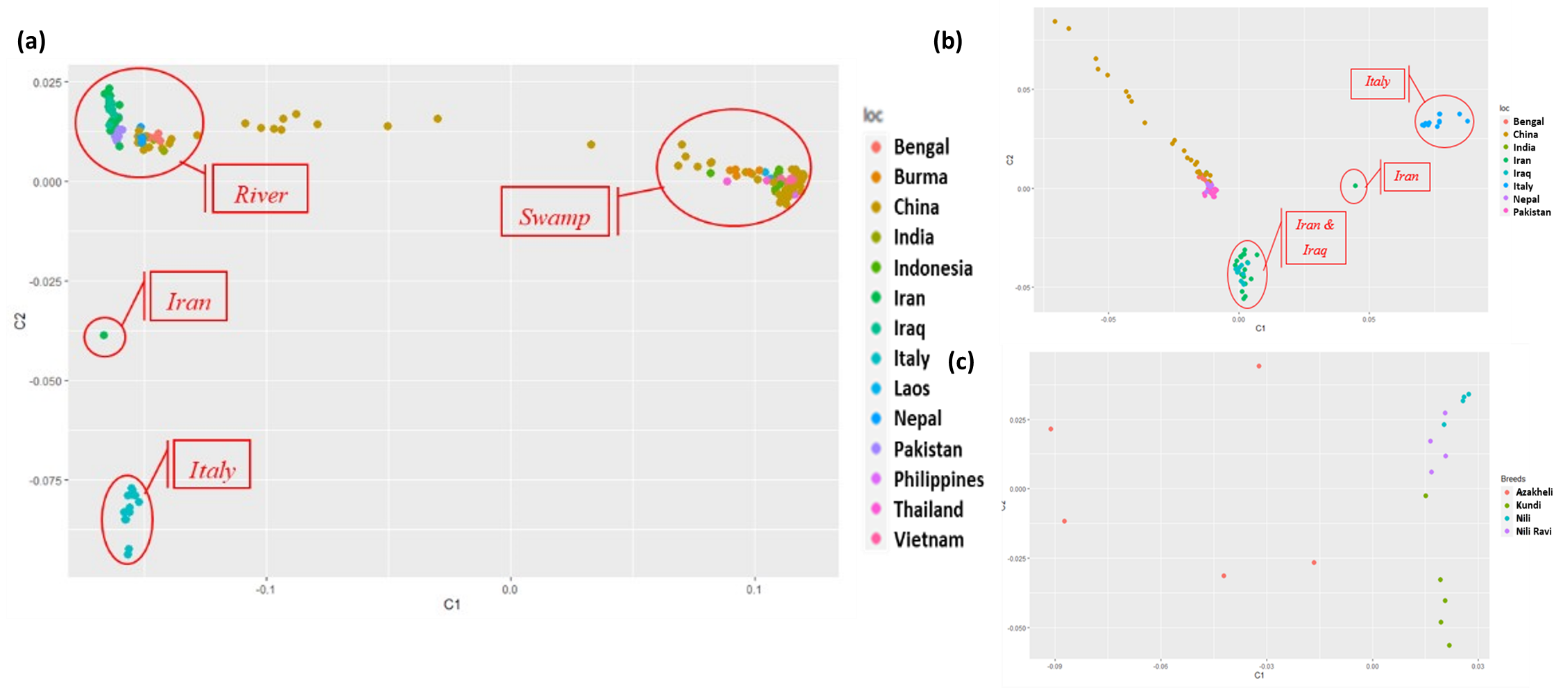


**Figure S1**: (a) Multi-Dimensional Scaling (MDS) analysis of River and Swamp type buffalo breeds showing IBS clustering for river type, swamp type, and for Iran and Italy buffalo breeds (b) MDS analysis of river type breeds only showing IBS clustering for Italy, Iran, Iraq and rest of the river breeds. (c) MDS analysis of buffalo breeds of Pakistan

**Figure S2**: Annotated summary of SNPs involving the Nili specific markers through SnpEff using river type water buffalo genome.

**Figure S3**: Annotated summary of SNPs involving the Nili Ravi specific markers through SnpEff using river type water buffalo genome.

**Figure S4**: Annotated summary of SNPs involving the Azakheli specific markers through SnpEff using river type water buffalo genome.

**Figure S5**: Annotated summary of SNPs involving the Kundi specific markers through SnpEff using river type water buffalo genome.
